# Experimental analysis of hydraulic fracturing for fracture network formation in coal beds

**DOI:** 10.1038/s41598-025-06745-9

**Published:** 2025-07-01

**Authors:** Jiwei Yan, Xiaoxia Song

**Affiliations:** 1 Key Laboratory of In-Situ Property-Improving for Mining of Ministry of Education, Taiyuan University of Technology, Taiyuan, 030024 Shanxi People’s Republic of China; 2 College of Mining Engineering, Taiyuan University of Technology, Taiyuan, 030024 Shanxi People’s Republic of China; 3 College of Geological and Surveying Engineering, Taiyuan University of Technology, Taiyuan, 030024 Shanxi People’s Republic of China

**Keywords:** Hydraulic fracturing curve, Hydraulic fracture, Multi-scale distribution, Control mechanism, Influencing factor, Coal, Natural gas

## Abstract

Understanding the distribution of multi-scale hydraulic fractures (HFs) is critical to improving coal bed methane (CBM) production. The HFs range from metres, centimetres to millimetres are revealed through coal mining face, X-ray CT, and stereoscope. The hydraulic fracturing curves can be categorised as descending, horizontal, ascending and fluctuating. While descending and horizontal types exhibited more effective hydraulic fracturing compared with ascending and fluctuating types. Macroscopic fractures are predominantly horizontal, vertical, X and T shaped. Closer to the CBM wellbore, the macroscopic fractures are more closely spaced and show greater connectivity with the bedding planes of various coal rock layers. During hydraulic fracturing, the fracturing fluid expands selectively along weak surfaces, such as joints in coal seams, leading to the formation of main fractures. Under high fracturing fluid pressure, HFs can penetrate various maceral specification layers and even propagate through coal gangue. Quartz sand embedded in the coal can trigger millimetre-scale HFs while remaining open. The development of multi-scale HFs is influenced by factors such as coal structure, the roof and floor strength, geo-stress, hydraulic fracturing design parameters, quartz sand and coal fines.

## Introduction

Hydraulic fracturing is employed to enhance the permeability of coal seams, and the distribution of hydraulic fractures (HFs) directly affects the effectiveness of this process. Understanding the control mechanisms of HFs is crucial for improving coal seam alteration and increase coal bed methane (CBM) production. During CBM production, methane diffuses from nanoscale pores and migrates into the wellbore through pores and fractures of varying sizes. Therefore, hydraulic fracturing aims to create a multi-scale inter-connected fracture network that facilitates methane migration. At present, research on HFs is mainly focused on laboratory experiments using small coal samples, resulting in a limited understanding of the distribution patterns of fractures in hydraulic fracturing.

Previous researchers have studied the distribution characteristics and formation mechanisms of HFs and proposed various physical and mathematical models to analyse their distribution. However, Deciphering the exact morphology of HFs on an engineering scale remains a challenge. The HFs exposed during coal mining offer a reliable means of directly observing their complex properties^1–4^. Warpinski et al. investigated the geometric distribution of HFs in coal mines, and concluded that their configuration is influenced by joints, faults, bedding planes and stress^5^. Diamond et al. compared fracture data from hydraulic fracturing of CBM wells with observations from coal mining, and found that the predominant fractures were vertically oriented, with some forming T-shaped configurations^6^. Horizontal fractures occur mainly at the coal seam-roof interface. Jeffrey et al. found that a small proportion of the vertical fractures are distributed parallel to and inter-laced with naturally occurring fractures in the coal^7^. Lv et al. revealed that horizontal fractures are influenced by interactions among the roof, floor, coal seams, macroscopic fractures between coal and rock strata and structural coal stratification^8^. Under continuous fluid action, discrete micro-fractures expand to form macroscopic fractures in sandstone samples^9^. The growth of the HFs network is a complex mechanical system involving rock deformation, fracture propagation and the inter-play of fluid-rock interactions.

The HFs in the coal can re-close under the pressure of the overlying strata during the flowback of the fracturing fluid. Therefore the proppant of quartz sand is used to keep the fractures open. The transported of quartz sand continuously impacts and abrades the fracture walls, generating coal fines. And their accumulation can obstruct the fractures^10,11^. Therefore, the intensity of quartz sand must be considered in relation to the formation closure pressure and the mechanical strength of the coal seam^12,13^. As quartz sand is embedded deeper into the coal seam, the effective opening of the HF decreases with increasing formation closure pressure^14^. Additionally, the conductivity of the fractures diminishes, lower sand concentration and softer coal seams due to greater embedding^15^. Understanding the characteristics of HFs and natural fractures is critical to optimising CBM production and enhancing reservoir modification^16,17^.

The propagation of vertical HFs is selective and influenced by the interaction of maximum principal stress and natural fractures. Horizontal HFs propagate in the direction of maximum principal stress, with their distribution and propagation being influenced by a number of factors, including the geo-stress field, coal structure, natural fractures, structural conditions and the rock properties of the roof and floor sections^18^. The magnitude of the minimum horizontal principal stress varies at different depths, resulting in different aperture sizes of the HFs^15,19^. A low in-situ stress differential results in the formation of a complex network of numerous small volume fractures, whereas a high in-situ stress differential results in large fractures with simpler morphologies^20^. Fracturing fluid presently follows the natural weak planes of the coal seam^21^. Brittle failure is more likely to occur in the coal seam with high mechanical strength. The HFs are prone to turning at the inter-faces of roof and floor of the coal seam. High displacement and sand ratios during fracturing can create complex volume fractures in the coal. But the accumulation of coal fines and quartz sand within the fractures can also alter the direction of fracture deflection^22^.

Previous studies have explored the distributional characteristics of HFs through theoretical and laboratory analyses. However, there has been less focus on the different scales of HFs and their control mechanisms. This article examines the evolution of HFs from metre to millimetre scales and identifies the factors that influence their formation. It elucidates the control mechanisms behind fracture development at different scales, providing valuable insights for understanding the coal bed reformulation by hydraulic fracturing.

## Geological background and experimental setup

### Overview of the study area

**Fig. 1:**
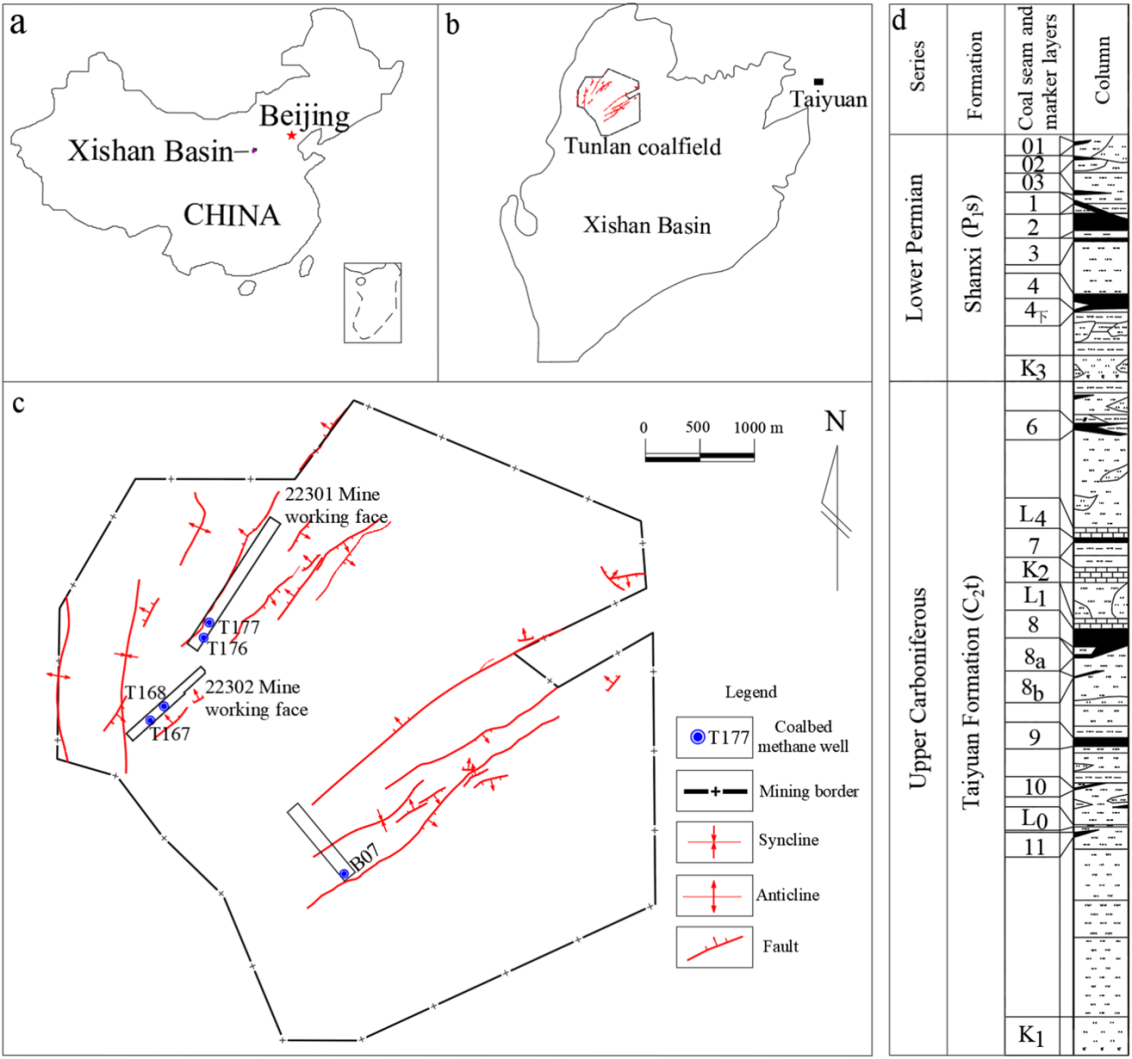
Location map and geological profile of the Tunlan coal mine.

The Xishan coalfield is situated in the central part of the Shanxi intra-plate orogenic belt within the North China Plate (Fig. 1a,b). The coal basin is almost triangular in shape and is bounded by faults. The Shizihe-Malan syncline traverses the central part of the coalfield in a south-north direction, accompanied by secondary folds that strike northwest and northeast, forming a compound syncline that is gentle to the east and steep to the west. The main coal-bearing strata in the region include the Taiyuan Formation of the Upper Carboniferous system and the Shanxi Formation of the Lower Permian system. The 2# and 8# coal seams are the main targets for CBM production in the area. This paper analyses the hydraulic fracturing characteristics of the 2# coal seam, which is located within the Shanxi Formation (Fig. 1d). The 2# coal seam is a coking coal primarily composed of semi-bright coal. The analysis examines the structure of the coal seam, as revealed by the 22301 (wells 176 and 177), 22302 (wells 167 and 168) and 12505 (B07 well) mine working faces (Fig. 1c), and examines the distribution pattern of fractures within the coal seam structure.

### Experimental conditions

The NanoVoxel-4000 ultra-high resolution X-ray three-dimensional computed tomography (CT) was employed to study the micro-fractures of coal. Prior to the computer radiography scanning, the samples were dried for 24 h at 80 °C. The parameters of the CT scanner were set to voltage 200 kV, current 300 mA, exposure time 0.70 s, number of images 2 and a pixel resolution of 1 × 1. The rotating table captured a total of 1440 images for each 0.25° angle rotation. The distribution of microfractures in different coal samples was obtained using reconstruction software. During the image reconstruction process, domain segmentation was performed to determine the distribution of the fractures. The methods used for domain segmentation were mainly adapted from the literature^20^.

For coal samples containing quartz sand, a Leica S9i stereoscope was used to examine the distribution of fractures and quartz sand. In order to preserve the original condition of the coal during sample preparation, samples were taken directly from the coal mass that containing quartz sand, with mirror clarity adjusted accordingly. The stereoscope was set to an optimum magnification of 40×, allowing detailed observation of fracture openings and the distribution of quartz sand and coal fines.

## Results

### Hydraulic fracturing stimulation outcomes

**Fig. 2:**
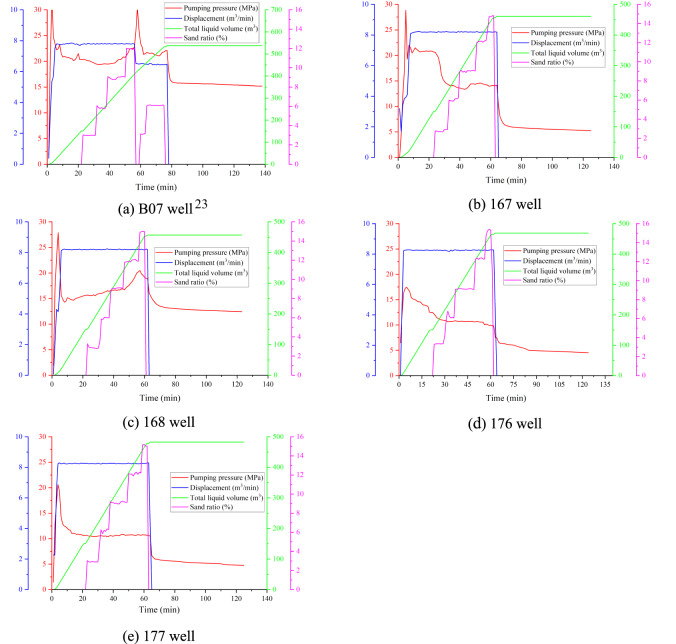
Hydraulic fracturing construction curves for the five CBM wells.

The fracturing fluid used is slick water, with pure quartz sand as the proppant, which is divided into fine and coarse sand with particle sizes of 0.45–0.90 mm and 0.80–1.20 mm, respectively. The quartz sand has excellent sorting properties, is flat to sub-oblate in shape, has both sphericity and roundness greater than 0.7 is free from impurities. Five CBM wells utilise the same hydraulic fracturing design. During the fracturing process, approximately 540 m^3^ of fracturing fluid is combined with 25 m^3^ of quartz sand, consisting of 20 m^3^ of fine sand and 5 m^3^ of coarse sand (Fig. 2).

The hydraulic fracturing design curves for the five CBM wells include pump pressure, displacement, total fluid volume and sand ratio (Fig. 2). Based on the sand addition process, the pump pressure curve can be divided into four stages: pre-fluid injection, sand addition, displacement fluid and pump shutdown. During the pre-fluid injection stage, HFs is generated primarily in the coal seam. The pumping pressure increases rapidly at the beginning and then decreases sharply after the coal fractures. There is a notable difference in fracturing pressure between the five CBM wells, even between adjacent wells such as 167 and 168, and 176 and 177, indicating significant heterogeneity within the coal seam that affects fracturing pressure. During the displacement stage, the pump pressure remains relatively steady. At the pump shutdown stage, the pump pressure curves for the five CBM wells show an initial steep drop followed by a gradual taper.

During the sand injection stage, the sand ratio starts at 3% and gradually increases to 12%. Initially, the pump pressure curve of the B07 well shows a gradual decrease. When the sand ratio reaches 6%, the pump pressure begins to rise steadily; 5 min after the sand ratio exceeds 12%, the pump pressure escalates abruptly, indicating severe sand blocking in the coal seam. The sand addition stops and displacement decreases. Once the sand blockage is removed, the pump pressure stabilises. Sand is added at rates of 3% and 6% until hydraulic fracturing is complete (Fig. 2a). For the 167 well, the pump pressure initially drops sharply during sand addition (Fig. 2b), indicating that the coal seam has good permeability, which facilitates sand addition. In contrast, the pump pressure of the 168 well increases slightly during sand addition (Fig. 2c), indicating that quartz sand and coal fines are mixing and accumulating, potentially plugging the fractures. As a result, the fractures gradually widen around the wellbore, and the filtration loss of the sand-carrying fluid remains relatively low. The fracturing pressure of the 176 well is the lowest of the five CBM wells at only 18 MPa, suggesting that natural fractures in the coal seams may be more pronounced near this well. The pumping pressures of the wells 176 and 177 remain stable throughout the sand addition stage (Fig. 2d,e), indicating that the injection volume of sand-carrying fluid matches the filtration loss, signifying effective fracture propagation during hydraulic fracturing.

The rapid rise in the fracture curve suggests that the extension of the HFs is either obstructed by proppants or coal fines or influenced by the heterogeneity of the coal seam. At this point the displacement of the fracturing fluid should be reduced. As a result, no significant main HFs are formed; instead, multiple small fractures occur simultaneously, connecting with the natural fractures in the coal seam and creating a radial network of fractures^24,25^. Diffusion of the fracturing fluid may result in inadequate fracturing effects on these small fractures. Conversely, the descending fracture curve indicates that the fracturing fluid is infiltrating along the main fracture. The main fracture communicates with some natural fractures, resulting in a gradual decrease in pumping pressure. Although the diffusion of the fracturing fluid is relatively extensive, the fracturing effect on small fractures may be limited due to the low pump pressure. The displacement of fracturing fluid can be appropriately increased to ensure the fracturing effect. A stable fracture curve indicates the presence of a significant number of natural fractures near the wellbore, with a relatively low opening pressure for these fractures^26^. As a result, the fracturing fluid can penetrate fractures of different sizes, facilitating the migration of CBM. Finally, the fluctuation fracturing curve indicates that the fracturing fluid infiltrates primarily along the natural fractures, resulting in a gradual increase during the initial stage. Once the fractures are adequately supported and opened, the pump pressure begins to decline^27^.

### Distribution characteristics of macroscopic fractures

**Fig. 3:**
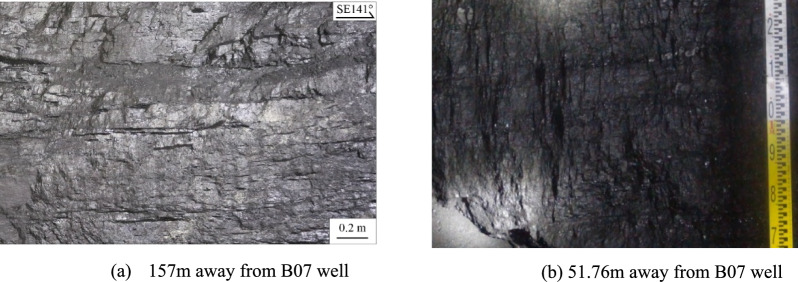
Coal seam unaffected by hydraulic fracturing^23^.

In coal seams not affected by hydraulic fracturing, clear horizontal bedding is evident. Different coal petrographic layers are arranged in parallel, and the coal structure retains its original shape, even under the influence of mining activities (Fig. 3). Under the influence of coal mining, the fractures in the coal seam are closed by the pressure of the overlying strata. By comparing the structure of the coal seam in the roadway, it can be seen that the original structure of the coal seam is complete without major structural fractures. There are no prominent vertical or oblique fractures traversing the coal seam. In contrast, coal seams affected by hydraulic fracturing show that the number of fractures developed increases as the distance from the CBM well decreases. The fracturing fluid can easily pass through the natural fractures in the coal seam. As the pressure of the fracturing fluid increases, the HFs continue to expand and form a main fracture in the coal bed. At the same time, the fracturing fluid filters into the secondary fractures surrounding the main fracture.

**Fig. 4:**
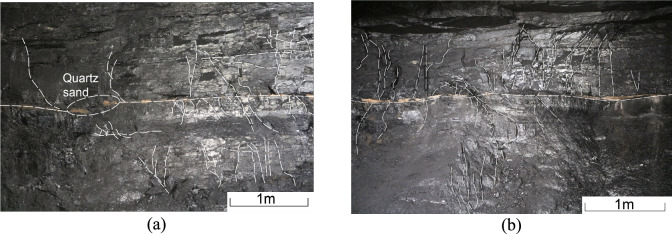
Distribution characteristics of fractures near the 168 well.

The kinetic energy of the sand-bearing fluid is relatively higher near the CBM well, facilitating its filtration into secondary fractures surrounding the main fractures and resulting in greater coal fragmentation. Observation of the coal mining face and combined with microseismic monitoring data during hydraulic fracturing of coalbed methane wells, has shown that the extension length of hydraulic fractures in the direction parallel to the maximum principal stress can reach 100–150 m, while the extension length in the direction parallel to the minimum horizontal principal stress can reach 70–90 m. The secondary fractures are distributed approximately vertically and are adjacent to the main fractures (Fig. 4). These secondary fractures are relatively dense, with some no longer extending as they expand; their lengths range from 6 to 100 cm, with spacings of 2 to 25 cm (Fig. 4a). While they are generally parallel, they can bend and converge during the extension process due to the influence of coal mechanical strength and natural fractures. Their opening is limited under low kinetic energy conditions of the fracturing fluid (Fig. 4b). The presence of coal fines within the fractures increases the migration resistance of the fracturing fluid, resulting in shorter extensions of secondary fractures and fewer fractures further from the main fractures. Only traces of secondary fractures can be observed, and their widths cannot be quantitatively characterised. Without quartz sand present, these secondary fractures will close as the fracturing fluid flows back.

**Fig. 5:**
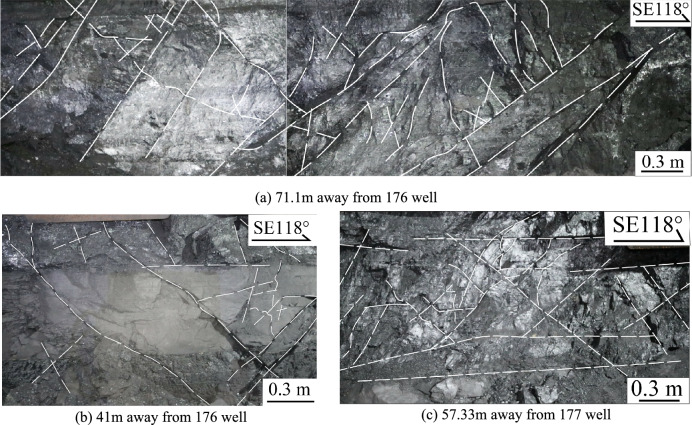
Distribution characteristics of fractures near the 176 and 177 wells.

The coal seams are typically semi-bright and have a fragmented structure. The fractures contain a significant amount of coal fines, and visible scratches on the coal surface indicate that the coal has been severely eroded by the fracturing fluid (Fig. 5a). There are few near-horizontal fractures, with most having an X-shaped configuration (Fig. 5b,c). Fractures extending through the gangue are visible (Fig. 5b), suggesting that the coal seam in this area has undergone intense hydraulic fracturing. These fractures maintain a stable orientation and traverse through different coal petrographic layers, arranged in an echelon pattern. The fracture surface appears straight and smooth, with some larger fractures exceeding 1 m in length (Fig. 5a). Fracture spacings range from 4 to 57 cm. The HFs are interconnected with horizontal bedding planes, forming a complex fracture network. Additionally, the fracturing pressure at the 176 well is relatively low, with pump pressure decreasing slowly after reaching the coal seam fracturing pressure. The shape and trend of the pump pressure curve indicates that numerous natural micro-fractures have developed near this well. These micro-fractures have a low fracture pressure, which facilitates the formation of a complex fracture network.

### Distribution of mesoscopic fractures

**Fig. 6:**
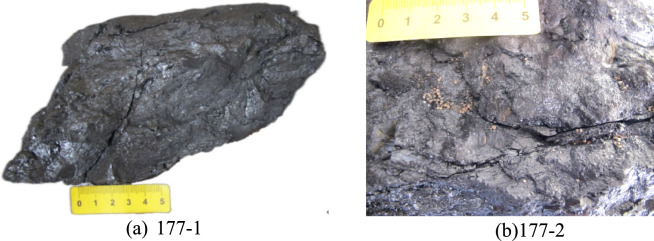
Coal samples collected near the 177 well.

Samples were collected from hydraulic fracturing coal seams exposed during coal mining and were significantly affected by hydraulic fracturing, particularly those coal samples located close to the main HFs (Fig. 6). These samples show clear characteristics of hydraulic fracturing and provide valuable insights into the effects of hydraulic fracturing on fractures of different sizes within the coal.

Figure 6 shows semi-bright coal with loose hand-tested strength. Samples 177–1 and 177–2 were taken from the 177 well at distances of 30.54 m and 5.24 m, respectively. The coal sample is grey black with external fractures and wrinkles. The bedding is indistinct, with a 0.12 cm wide fracture surrounded by numerous small secondary fractures (Fig. 6a). The coal sample is fragmented and shows clear fracture marks. HFs containing quartz sand have a greater degree of opening (Fig. 6b) and the surface shows evidence of fracturing fluid flushing, covered by a significant amount of coal fines and quartz sand. During fracturing, the quartz sand has a low transport velocity in the fracture and its friction with the fracture wall becomes stronger, leading to the coal fines filling the fracture. This in turn leads to the mixing and accumulation of quartz sand and coal fines in the fracture, resulting in a reduction in the extension length of the hydraulic fracturing fracture and a weakening of its ability to expand.

**Fig. 7:**
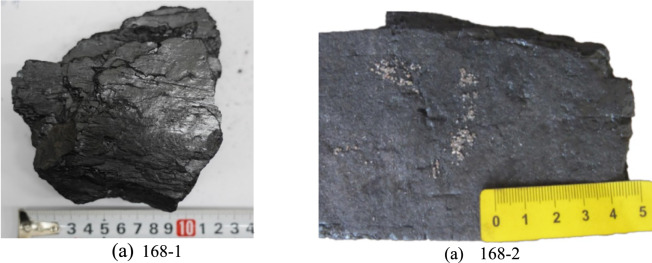
Coal sample collected near the 168 well.

Samples 168–1 and 168–2 were collected from the 168 well at distances of 24.5 m and 8.7 m, respectively (Fig. 7). As the effect of hydraulic fracturing on coal structure increases from weak to strong, significant differences in coal structure are observed. Figure 7a shows semi-bright coal, which has relatively high strength and allows some primary bedding planes to be distinguished. The irregular HFs in this sample have a considerable degree of opening. In contrast, Fig. 7b presents semi-dark coal with lower strength and indistinct bedding planes, containing a considerable amount of adherent quartz sand after crushing, together with visible large fractures. When hydraulic fracturing is carried out in deep coal seams, higher strength quartz sand may be required, and the flow rate of the fracturing fluid may need to be greater, which is conducive to coal fracturing and the expansion of the hydraulic fracturing fractures.

### Microscopic CT scanning analysis

**Fig. 8:**
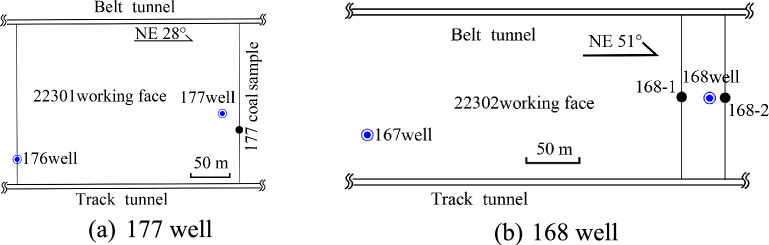
Location of coal samples.

**Fig. 9:**
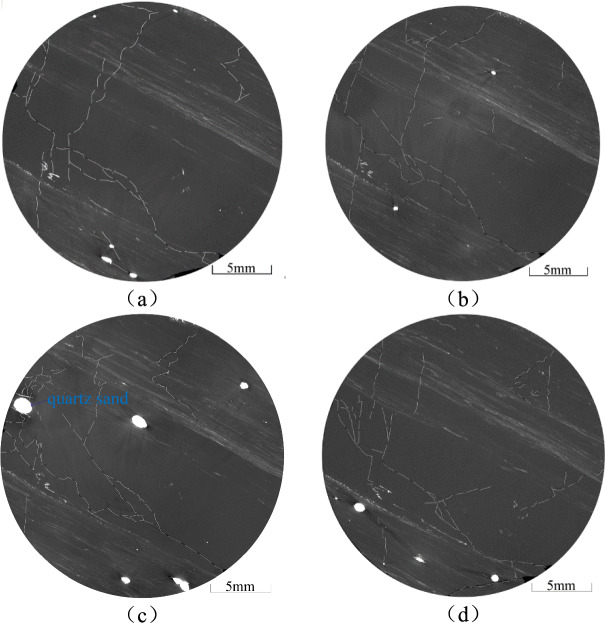
Microscopic CT scan of coal sample 177-1.

**Fig. 10:**
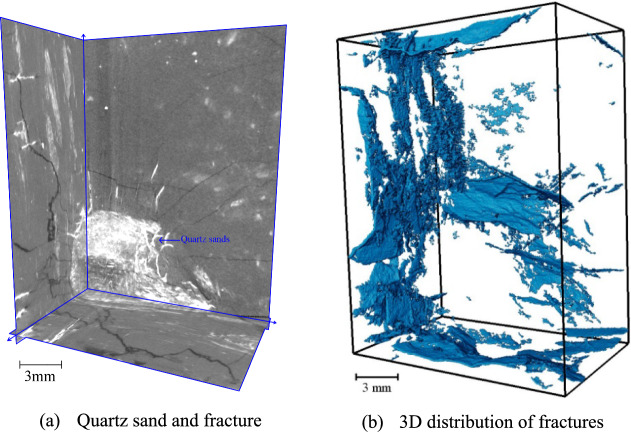
Distribution characteristics of micro-fractures in coal near B07 well^23^.

**Fig. 11:**
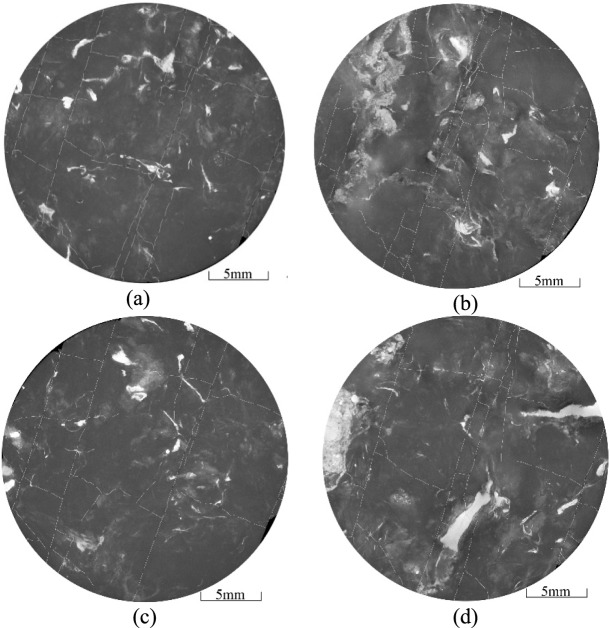
Micro-fractures observed in the 168-1 coal sample.

The location of the coal samples is shown in Fig. 8. A *φ* 25 mm × 50 mm coal sample was drilled along the parallel bedding direction using wire cutting to minimise the formation of new fractures during preparation. The samples were then subjected to micro-CT analysis to extract the spatial distribution of fractures and quartz sands. The two-dimensional sections of the coal sample were filtered and pre-processed (Figs. 9, 10, 11). In these images, fractures in the coal are represented by short white lines, while brighter white areas indicate the presence of quartz sand. The microfractures generated by coal metamorphism are mainly caused by shrinkage of the coal matrix due to water loss, with a relatively regular distribution, and there is no evidence of fracturing fluid compression or quartz sand friction around the fractures. The fractures caused by geological structures have an obvious directionality and no quartz sands.

The primary parallel bedding of the coal sample is not readily apparent (Fig. 9a–d). However, the various fractures exhibit a cross-distribution pattern after hydraulic fracturing, characterised by large openings and good connectivity. Irregular microscopic fractures are created by the filtration of the fracturing fluid and additional coal fractures are created by the embedding of quartz sand. Smaller secondary fractures are found around the larger fractures (Fig. 9a–d). Fractures adjacent to the quartz sand are dense and have a high degree of openness. Where there is a significant accumulation of quartz sand particles, radial fractures form around them (Fig. 9c, Fig. 10). As the quartz sand becomes embedded in the coal, it compresses the coal, causing further fracturing. This process contributes to the formation of a more complex network of natural fractures and HFs within the coal (Fig. 9b). Straight fractures can develop in areas of uniform coal petrography (Fig. 9c, d). The primary bedding planes in the coal are weak, causing tensile fractures as the fracturing fluid infiltrates these weak zones. As the fracturing fluid is discharged, the pore pressure within the fracture decreases, increasing the effective stress due to the overlying rock on the coal seam. The absence of quartz sand in the fractures can cause them to close.

**Fig. 12:**
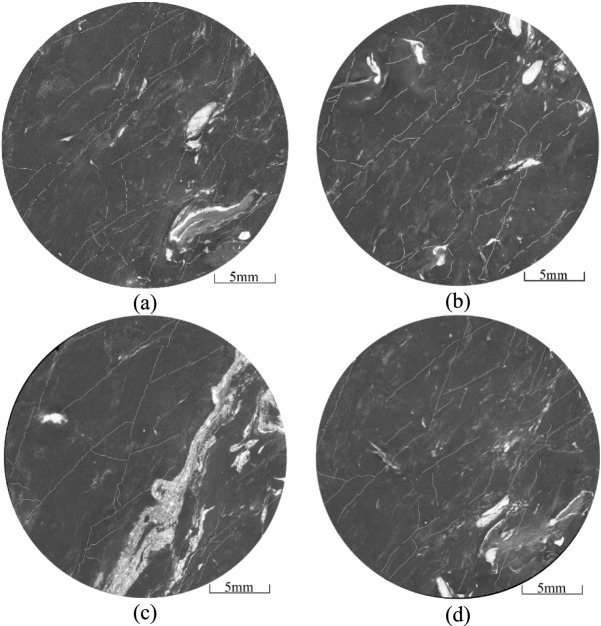
Micro-fractures observed in the 168-2 coal sample.

The complexity of the HFs in the coal decreases with distance from the CBM wells, where the pressure of the fracturing fluid on the coal and quartz sand is greater. The number of newly formed micro-fractures increases with the amount of quartz sand embedded in the coal (Fig. 11), further contributing to the complexity of the fracture network. As the degree of coal damage increases, so does the production of coal fines within the fractures. The embedding process of the quartz sand can result in continuous abrasion of the fracture walls, resulting in additional coal fines and increased fragmentation of the quartz sand. And the supporting effect of quartz sand on hydraulic fracturing fractures weakens, resulting in a decrease in the opening of hydraulic fracturing fractures and a decrease in the flow capacity of fractures. These coal fines and smaller quartz particles can accumulate and block the fractures (Fig. 11, Fig. 12). Due to the small aperture and weak connectivity of the secondary fractures, when fracturing fluid enters the secondary fractures, its kinetic energy is very low and it is unable to carry quartz sand into these secondary fractures.

## Discussion

### Impact of geological conditions on HF

**Fig. 13:**
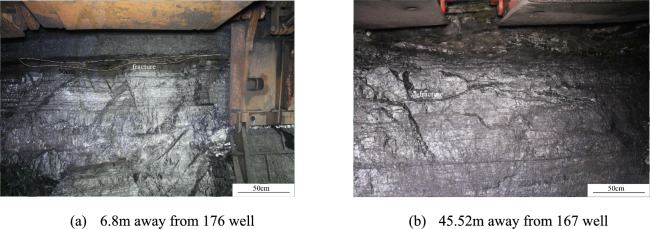
Distribution characteristics of fractures.

In the 176 well, the quartz sand is predominantly within the main horizontal fractures, accompanied by several secondary fractures in the lower part (Fig. 13a). The roof of the coal seam has high mechanical strength, making it difficult for the fracturing fluid to penetrate and expand upwards. Consequently, the fluid migrates mainly downwards, with limited upward movement. In contrast, the lower coal seam within the main fracture has lower mechanical strength, facilitating the transport of the fracturing fluid^28^. Hydraulic fracturing in coal seams produces predominantly horizontal and X-shaped fractures, which are largely influenced by the original bedding planes of the coal seam (Fig. 13b). A significant number of secondary fractures are distributed around the main horizontal fractures, creating a volumetric fracture network that enhances reservoir transformation. However, the main vertical fractures are relatively limited in extent, limiting their ability to effectively increase CBM production.

In primary structural coals, the fracturing fluid expands primarily along the face cleat and/or bull cleat. In contrast, in cataclastic and fragmented coals, tectonic influences lead to the formation of numerous fractures, causing the fracturing fluid to expand predominantly along these existing fractures. As tectonic deformation increases, the coal structure becomes increasingly fractured. Mylonitic coal has very low mechanical strength, resulting in underdeveloped fractures. This contributes to a high concentration of coal fines during the hydraulic fracturing process. The pre-existing fractures can cause fracturing fluid to leak, and fracturing fluid will migrate along these fractures, causing the hydraulic fractures to turn and not extend as originally designed. Therefore, when selecting a coalbed methane well site, it is important to avoid areas with developed natural fractures as much as possible.

**Fig. 14:**
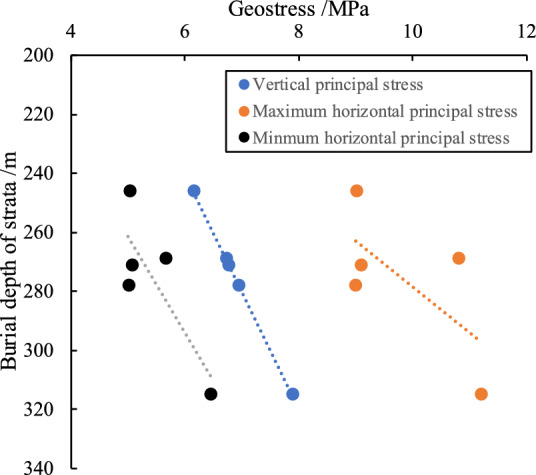
Variation of geo-stress in the Xishan coalfield (adapted from^29^).

In the Xishan coalfield, the dominant underground stress is the horizontal principal stress, which is significantly influenced by the structural stress^29^. The maximum horizontal principal stress is oriented in the north-west and north-east directions. For burial depths less than 250 m, the magnitude of structural stress follows this order: maximum horizontal principal stress (σ_H_), minimum horizontal principal stress (σ_h_) and vertical principal stress (σ_v_), i.e. σ_H_ > σ_h_ > σ_v_ . At burial depths between 250 and 600 m, the order shifts to σ_H_ > σ_v_ > σ_h_. The geo-stress at the five measuring points in the Tunlan mine is predominantly oriented between N6°E and N87°E, with burial depths ranging from 246 to 315 m. In this tectonic area, the stress field is characterised by the principal stress orientation of σ_H_ > σ_v_ > σ_h_ (Fig. 14).

In this area, hydraulic fracturing is primarily influenced by formation stress, the mechanical properties of the coal itself and those of the surrounding rock. These factors combine to influence the formation of vertical fractures. The dense nature of the rock and the high mechanical strength of the roof and floor present significant challenges to the extension of HFs from the coal seam into the surrounding strata. As a result, hydraulic fracturing causes fractures near the CBM well to expand upwards. When these fractures encounter weak bedding planes, they tend to turn and expand horizontally, intersecting the bedding planes at an angle. Consequently, the morphology of HFs is characterised by horizontal, vertical and T-shaped fractures^22,23,28^.

### Parameters of hydraulic fracturing construction

Hydraulic fracturing requires a constant pump pressure and displacement to create optimal conditions for the fracturing fluid or sand carrying fluid within the main fractures. The kinetic energy of the fracturing fluid decreases in the secondary fractures, and the presence of coal fines further impedes fluidity, reducing the dimensions of these secondary fractures. Stress shadowing can occur between parallel secondary fractures and horizontal fractures as they open, causing the coal between the secondary fractures to become denser after displacement, inhibiting their development. When displacement is low, quartz sand is deposited in the main fractures with shorter migration distances. Therefore, the pump pressure influences the initiation and expansion of the HFs, while the amount of displacement influences both the expansion of these fractures and the migration and placement of quartz sand within them.

As pumping pressures and displacements increase, the length, width and total volume of the main HF increases and more natural fractures are activated. Higher pump pressures and larger displacements boost the flow rate of fracturing fluid into the fractures, allowing greater quantities quartz sand to be transported and facilitating its migration within the main HF^30^. Significant pre-flushing enhances the extent of HF by elevating fluid pressure, which generates primary fractures, facilitates communication with more bedding planes and promotes quartz sand placement towards the distal ends of the fractures. To ensure even distribution within the HF, it is essential to increase the amount of quartz sand. Larger quartz sand particles may have difficulty being transported through narrow secondary fractures, while finer quartz sand can enter these secondary fractures, increasing the volume of supported fractures. However, these secondary fractures tend to recline after the flowback of the fracturing fluid. Low-viscosity fracturing fluids are advantageous for penetrating small fractures, helping them to open and widen^31^.

If the displacement of the fracturing fluid is substantial, it can rapidly initiate and expand the main HFs. Simultaneously, the fracturing fluid continuously penetrates the secondary fractures near the main HFs. As the pressure of the fracturing fluid increases, it promotes infiltration into smaller secondary fractures. Near the wellbore, the pressure remains relatively high, leading to a more extensive extension of secondary fractures in this area. However, as the fracturing fluid moves further from the wellbore, significant pressure loss occurs, and the kinetic energy available for migration into the secondary fractures decreases. This results in a reduced effect on the expansion of these secondary fractures.

### Effects of quartz sand and coal fines on fracture behaviour

**Fig. 15:**
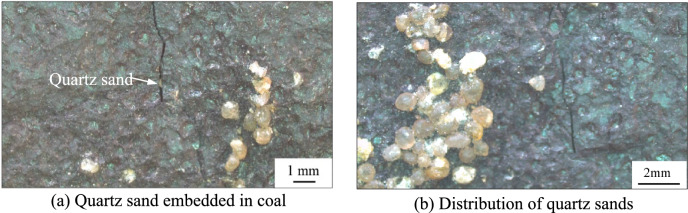
Distribution characteristics of quartz sand and fractures.

**Fig. 16:**
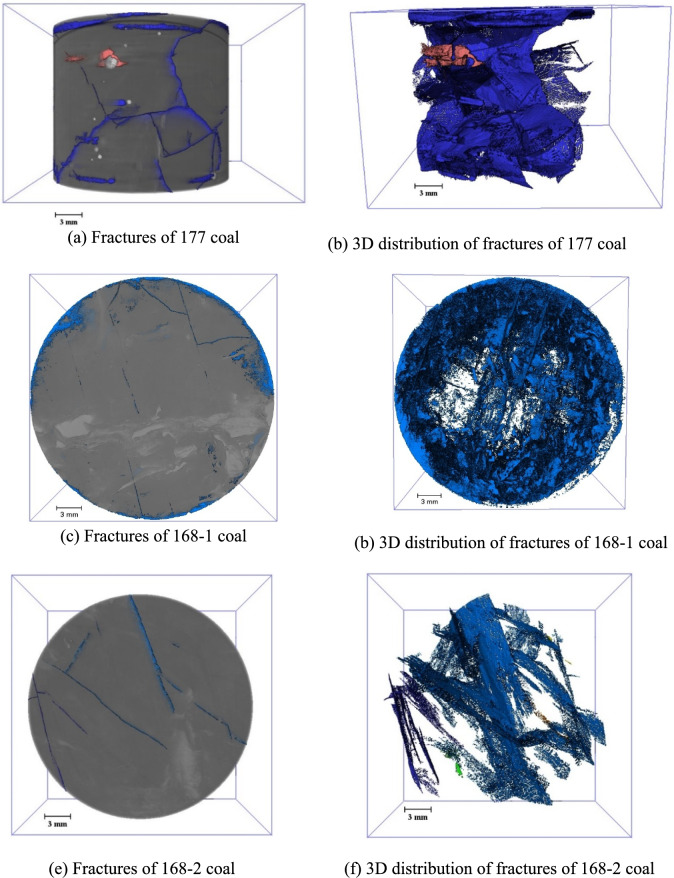
Fractures generated by the embedding of quartz sand in coal.

The embedding of quartz sands in the coal reduces its ability to support fractures (Fig. 15a). If a single quartz sand particle occupies a fracture, it can easily become closed or have only a small opening due to the wetting effects of the fracturing fluid and the influence of geostress. Consequently, the support capacity of a single grain of quartz sand for coal fractures is limited. Fractures close if they are not filled with quartz sand (Fig. 15b). As quartz sand is transported within the fractures, it embeds in the coal under high closure pressure. With increasing accumulation, the pressure exerted by the quartz sand on the coal escalates, potentially damaging the coal as the depth of embedment increases. However, when multiple quartz sand particles accumulate, they can support each other, effectively maintaining the fracture width and facilitating fluid migration (Fig. 16). This interaction can enhance the permeability of the coal seam^32^.

During the transport of quartz sand, continuous rubbing and collisions with the fracture walls can cause the particles to break and lose their roundness. This degradation increases the resistance to subsequent movement of the quartz sand. The increased surface area of the broken quartz particles makes it easier for coal fines to be adsorbed onto them, which can lead to blockages in the micro-fractures. Quartz sands are typically transported under strong hydrodynamic conditions, often forming sandbars within the main fractures. In contrast, quartz sand transport in secondary fractures, where hydrodynamic conditions are weaker, is less effective. As a result, only a few quartz particles may become embedded in the coal mass, where they can support and create micro-fractures (Fig. 16a,b).

Hydraulic fracturing results in the development of microfractures, which show good connectivity between different microfractures (Fig. 16a–f). However, coal fines are produced during this process due to coal fragmentation, friction from embedded quartz sand and flushing of fracturing fluid through the coal. The sand carrying fluid, with its high hydrodynamic intensity, can transport these coal fines within the HFs. As the coal fines migrate and collide with the fracture walls, their accumulation increases. This accumulation leads to a reduction in the aperture of the HFs, resulting in a rapid decrease in the hydrodynamic intensity of the sand-carrying fluid. Consequently, the accumulation of coal fines significantly obstructs the fractures, and prevents their expansion towards the far end.

## Conclusions

Through underground observations and laboratory tests, we have analysed the distribution characteristics of HF networks at different scales. The following conclusions can be drawn:

1. The macro-HFs in coal intersect with natural fractures, which can take a variety of forms, including horizontal, vertical, X-shaped and T-shaped. Some macro-HFs are able to extend through the gangue. In contrast, micro-HFs are distributed in a radial pattern around the quartz sand.
2. Quartz sand is mainly present in the main horizontal fractures, with less secondary fractures surrounding them. Embedding quartz sand in the coal increases the support for micro-HFs. As the effective stress on the fractures increases, the fractured quartz sand can fracture, producing smaller particles that are more likely to absorb coal fines.
3. The coal has been hydraulically fractured, resulting in numerous macro and micro fractures distributed in a tortuous manner. However, fractured quartz sand particles and coal fines can block these fractures. Conversely, coal that has not undergone hydraulic fracturing is mainly composed of primary parallel bedding planes.
4. The distribution of HFs is closely related to several factors, including coal structure, mechanical properties of the coal, roof and floor strength, stress field, natural fractures, fracture construction methodology, fluid viscosity and the presence of coal fines.

## Data Availability

All relevant data are within the paper. The datasets are available from the corresponding author upon reasonable request.
